# Intradermal influenza vaccination in complete remission cancer patients: molecular insights

**DOI:** 10.1186/2051-1426-1-S1-P197

**Published:** 2013-11-07

**Authors:** Davide Bedognetti, Maria Libera Ascierto, Marimo Sato-Matsushita, Elena Gugiatti, Carlotta Massucco, Simonetta Zupo, Antonio Di Meglio, Chiara Dellepiane, Mario Roberto Sertoli, Omar Racchi, Enrico Balleari, Valeria De Giorgi, Michele Sommariva, Paolo Durando, Manlio Ferrarini, Roberto Cacciani, Nicoletta Provinciali, Rocco Iudici, Cristiano Alicino, Ena Wang, Filippo Ansaldi, Francesco M Marincola, Andrea De Maria

**Affiliations:** 1 DTM, NIH, Bethesda, MD, USA; 2 IRCCS San Martino-Ist, Genova, Italy; 3 Sidra Medical and Research Centre, Doha, Qatar

## 

We previously showed that long-lasting complete remission (CR) non-Hodgkin lymphoma (NHL) patients treated with rituximab-containing chemotherapy have an attenuated antibody response to virosomal (Bedognetti et al, J. Immunology, 2011) or MF-59 adjuvanted (Bedognetti et al, Blood, 2012) seasonal (or pandemic) influenza vaccine (as compared with healthy controls), associated with persistent CD27+ Memory B cell depletion and hypogammaglobulinemia. Here, we evaluated humoral and innate response to trivalent intradermal vaccination in NHL in CR previously treated with rituximab-containing chemotherapy (at least one year before vaccine administration), RIT group, and in CR cancer patients treated with chemotherapy without rituximab (at least one year before vaccine administration), Non-RIT group. Intradermal administration was chosen considering its promising data, compared to conventional intramuscular route, in terms of immunogenicity and safety. Humoral response was assessed by hemagglutinin inhibition assay on sera collected at time 0 (just before vaccination) and at time 28 (four weeks after vaccination). Innate response was assessed by whole-genome gene expression analysis (Affymetrix Humane Gene ST 1.0) on PBMC collected at time 0 and at time 1 (24 hours after vaccine administration). Patients treated with rituximab-containing chemotherapy had, overall, a lower antibody response, compared to patients treated with chemotherapy alone. Overall, intradermal vaccination induced dramatic changes in gene-expression profile already one day after vaccination. These changes underline the activation of IFN stimulated genes (eg, STAT1, STAT2, CXCL10, IDO1, GBP1) and modulation of NK-associated transcripts. In addition, pathway and gene-enrichment analysis show that RIT and non-RIT groups have different quantitative and qualitative transcriptomic changes 24 hours after vaccination administration. Concordantly with antibody-titer, the innate response was more intense in Non-RIT group compared with RIT group.

